# Auxin mediated synthesis of gold nanoparticles: a novel approach to enhance shoot and root growth in pearl millet

**DOI:** 10.3389/fpls.2025.1621007

**Published:** 2025-09-11

**Authors:** Sahil, Anis Ahmad Chaudhary, Sonia Sorout, Kushi Yadav, Mohamed A. M. Ali, Fehmi Boufahja, Vikram Kumar, Devendra Jain, Kumar Sambhav Verma

**Affiliations:** ^1^ Amity Institute of Biotechnology, Amity University Rajasthan, Jaipur, Rajasthan, India; ^2^ Department of Biology, College of Science, Imam Mohammad Ibn Saud Islamic University (IMSIU), Riyadh, Saudi Arabia; ^3^ Amity Institute of Biotechnology, Amity University Uttar Pradesh, Noida, Uttar Pradesh, India; ^4^ Amity Institute of Pharmacy, Amity University Rajasthan, Jaipur, Rajasthan, India; ^5^ Maharana Pratap University of Agriculture & Technology, Udaipur, Rajasthan, India

**Keywords:** nanobionics, gold nanoparticles, indole-3-butyric acid, indole-3-acetic acid, phytotoxicity

## Abstract

**Introduction:**

Pearl millet, a staple in arid areas, usually suffers from inadequate root development due to environmental stressors. Specifically, using nanotechnology, gold nanoparticles (AuNPs) provide a revolutionary way to improve plant development.

**Methods:**

Auxins, specifically Indole-3-acetic acid (IAA) and Indole-3-butyric acid (IBA), were used as stabilizing and reducing agents in the synthesis of AuNPs. UV-Vis, FTIR, SEM, zeta potential, and DLS studies were used to characterize the nanoparticles. Their effect on pearl millet seedlings was evaluated by in vitro tests.

**Results:**

The control seedlings had shoot and root lengths of 2 cm and 3.5 cm, respectively. With IAA-AuNPs, shoot and root lengths increased to 5.25 cm and 6.75 cm, while with IBA-AuNPs, they increased to 4.75 cm and 7.75 cm. No phytotoxic effects were discovered.

**Discussion:**

Pearl millet development was markedly enhanced by IAA- and IBA-stabilized AuNPs, indicating their potential as safe and efficient growth enhancers in situations impacted by stress.

## 1 Introduction

Nanotechnology has rapidly developed as a field, growing from a dream initiated by Richard P. Feynmans groundbreaking lecture, “Theres Plenty of Room at the Bottom”. One of the most important creations to have arisen from it is the nanoparticle (NP), a material with at least one dimension that is less than 100 nm (Patel et al., 2021). Nanoparticles have been utilised in many ways, notably in agriculture, although varying uses have appeared everywhere. Nanoparticles vary in structure and can be classified as geometric zero, one, two, or three dimensions. Nanobionics is a juncture between nanotechnology and biotechnology, and engineered nanomaterials have emerged for use and potential in agriculture to influence crop productivity (Altammar, 2023). In nanobionics, materials can act, with a degree of precision, as a “nanocarrier” for materials (such as genes, nutrients, and agrochemicals) or allow for controlled release of active materials, thus increasing effectiveness and efficiency for farming, and the potential for decreased environmental impact. A review of the literature reveals a gap in reliable data on their biological effects, with some studies suggesting potential phytotoxicity due to variations in size and concentration (Santás-Miguel et al., 2023). While nanoparticles appear promising, we still do not fully understand the interactions between the size of NPs and plants. Therefore, it is essential to understand the potential environmental trade-offs, as the same nanoparticle can vary in effectiveness and toxicity depending on its concentration or shape (Ameen et al., 2021).

AuNPs are among metallic nanoparticles that have increased biocompatibility and decreased phytotoxicity. (Raza et al., 2024). AuNPs possess unique optical and chemical properties such as surface plasmon resonance (SPR), fluorescence quenching ability, and ease of biofunctionalisation. AuNP applications in agriculture have yielded promising results, resulting in up to 35% chlorophyll content, antioxidant levels have increased greater than other pesticides, and root and shoot growth for maize, asparagus, and *Brassica juncea* increased, ranging from 30-50% in root elongation (Kumalasari et al., 2024). To promote sustainable development in agriculture, a new form of gold nanoparticle (AuNPs) has been developed using naturally occurring plant hormones—Indole-3-Acetic Acid (IAA) and Indole-3-Butyric Acid (IBA) (Butova et al., 2023). These hormones aid in overall plant health by promoting root growth and shoot elongation. In another way, these plant hormones become reducing agents to form and develop AuNPs. The dual benefit of using hormones is that they allow AuNPs to slowly provide the same hormones to the plant, increasing growth efficiency and decreasing the waste of traditional hormone treatments.

This study evaluates the development of AuNPs through the auxin-mediated process. It examines whether the immobilisation will enhance the early growth stages of *P. glaucum* (Pearl millet), a critical cereal crop for arid agricultural regions. The initial data demonstrated that the auxin-AuNP treatments resulted in a significant root length increase (41%), with shoot length (28%), and the leaf surface area increased (33%) over the untreated controls.

The study was designed to analyse not only the individual effects of each factor but also how priming duration interacts with various hormone combinations in *P. glaucum*. The findings demonstrate that auxin-stabilised gold nanoparticles offer an effective and environmentally friendly method to enhance crop growth under challenging agricultural conditions over time.

## 2 Methodology

### 2.1 Reagents

Indole-3-acetic acid (IAA) and indole-3-butyric acid (IBA) were used as sources of reducing agents for Gold (III) chloride trihydrate (HAuCl_4_) with KOH to maintain the pH. They were acquired from Sigma-Aldrich Co., Ltd, USA. Milli-Q water was obtained using a Millipore system (USA), having 18.2 MΩ ionic purity.

### 2.2 Preparation of gold nanoparticles

AuNPs were prepared using 1 mL of gold chloride (HAuCl4) solution 1mM as the source of gold, 860ul of potassium hydroxide (KOH) solution 10mM with IAA or IBA, and the final volume was adjusted to 50 mL using deionised water. To make the solution, 43 mL of deionised water was heated to 60°C in a magnetic stirrer, then KOH was added drop by drop. The IAA or IBA was then added (one or the other, depending on the experimental grouping), whereby the HAuCl_4_ was added drop by drop to stop any aggregation of the nanoparticles. The solution turned from clear to red wine, indicating the stabilisation with IAA or IBA creating the colloidal AuNPs (Maciej Serda et al., 2013; Ghorbani, 2015). The addition of IAA or IBA commenced the reaction, and following almost 1 hour, the wine-red colour was the endpoint of the reaction. The solution was then stirred briefly for an additional 3-4 hours for dispersion (Oliveira et al., 2023).

## 3 Characterisation of IAA- and IBA-stabilised AuNPs

Characterisation and analysis of nanoparticles are essential tasks that provide detailed statistical and structural data on their shape, size, size distribution, dispersion, surface charge, and overall physicochemical properties. The IAA- and IBA-stabilised gold nanoparticles were characterised and used for *in vitro* experimental studies.

This study recorded UV-Vis spectra of AuNPs synthesised using IAA and IBA, along with solutions of IAA and IBA as a control, using a UV-Vis Spectrometer (Thermo Scientific Multiskan GO). To remove excess stabilising agents, IAA- and IBA-stabilised AuNP solutions were centrifuged at 10,000 rpm for 10 minutes at 15°C. The resulting nanoparticles were then diluted in ultrapure distilled water for further analysis. To avoid overloading, the IAA- and IBA-stabilised AuNPs were diluted in a 1:20 ratio with deionised water before being subjected to scanning electron microscopy (SEM) analysis. After that, 2 µL AuNPs were placed onto a glass slide, covered and allowed to air dry at ambient temperature. After coating it with gold, FE-SEM (TESCAN MIRA, Brno, Czech Republic) operated at an accelerating voltage of 25 keV with an SE detector, which was used to investigate the sample. The hydrodynamic diameter of the nanoparticles was measured by dynamic light scattering (Litesizer 500 Anton Par, Austria), using a backscatter detector with an angle of 15°, 90°, 175° at 25°C. Using the same apparatus, the zeta potential representing the total charge that the particles in a given medium have acquired was also measured at 25°C.

Fourier-transform infrared (FT-IR) spectroscopy (ABB MB 3000) investigated the interactions between IAA, IBA, and gold nanoparticles. The stabilised AuNPs were centrifuged at 14,000 RPM for 10 minutes to prepare the samples. The resulting pellet was ground into a fine powder using potassium bromide (KBr). This powder was analysed using FTIR over 400-4000 cm^-^¹. The transmission percentages were recorded and compared with pure IAA and IBA spectra, which served as control samples.

## 4 Seed priming studies of IAA -and IBA-stabilised AuNPs

### 4.1 Plant materials and growth conditions

All Seed Priming experiments were conducted under controlled conditions in a culture room maintained at 25 ± 2°C, with uniform light (1000 Lux) provided by fluorescent tubes on a 16/8-hour light/dark cycle using pearl millet seeds as a plant material.

### 4.2 Application and assessment of IAA- and IBA-stabilised AuNPs through seed priming

The rooting ability of IAA- and IBA-stabilised gold nanoparticles (AuNPs) in pearl millet was investigated compared to controls using *in vitro* tissue culture—initial control experiments involved treating *in vitro* seeds with deionised water and a combination with hormones. In the experiments, solutions of AuNPs synthesised using IAA and IBA were used at the same concentration during nanoparticle synthesis. For the *in vitro* studies, 10 mL of each hormone-stabilised gold nanoparticle solution was combined with 10 mL of double-distilled water containing 700 µL of IAA and 800 µL of IBA to prepare the treatment mixture.

Seeds of pearl millet were immersed (1 inch deep) in separate 10 ml vials containing AuNPs IAA-stabilised (set d), AuNPs IBA-stabilised (set e), kept at a magnetic stirrer for 0, 1, 3 and 6 hours, respectively. Control treatments included seeds treated with native IAA (set b) and IBA (set c) as positive controls, and water-treated explants (set a) as a negative control for 0, 1, 3 and 6 hours, respectively (Sevik and Guney, 2013). Further, 5 seeds were placed on petri plates for each set. 2 mL of water was added for each set. Later, the plates were placed in a plant tissue culture chamber, under controlled conditions maintained at 25 ± 2°C, with uniform light (1000 Lux) provided by fluorescent tubes on a 16/8-hour light/dark cycle and observed from germination to till sufficient root and shoot length.

### 4.3 Biochemical studies

#### 4.3.1 Antioxidant enzyme activity

Used the method to extract the enzyme samples. Microgreens (0.1 g) were extracted with 10 mL of 0.1 M phosphate buffer solution (PBS; pH 7.0). The sample was centrifuged at 16,000 rpm, 4°C for 20 minutes (Al-Zuhair et al., 2016).

#### 4.3.2 Superoxide dismutase

The SOD content was determined following the method of Beauchamp and Fridovich (1971) with minor modifications. The extract (0.1 mL) was mixed with Na_2_CO_3_.NaHCO_3_ buffer (50 mM, pH 10.2), nitroblue tetrazolium chloride (1 mM), xanthine (4.0 mM), ethylene diamine tetraacetic acid (3.0 mM), bovine serum albumin (0.15%), and xanthine oxidase (0.1 mL). The mixture was incubated at 30°C for 20 minutes, followed by adding CuCl_2_ (8 mM). The absorbance was recorded at 560 nm (Beyer and Fridovich, 1987).

#### 4.3.3 Catalase

The CAT content was determined according to the method suggested by Pukacka and Ratajczak (2005). The extract (0.5 mL) was mixed with H_2_O_2_ (40 mM) and 0.1 M PBS (2 mL). The absorbance was recorded at 240 nm (Pukacka and Ratajczak, 2005).

#### 4.3.4 Ascorbate peroxidase activity

The APX activity was determined using the method described by Nakano and Asada. The extract (0.1 mL) was mixed with H_2_O_2_ (0.4 mM) and 50 mM PBS containing 0.5 mM ascorbic acid (1.9 mL). The absorbance was recorded at 290 nm (Kumar, 2022).

#### 4.3.5 Statistical analysis

All the experiments were carried out in triplicate, and two-way ANOVA was performed to analyse the effects of IAA and IBA stabilised AuNPs on root and shoot growth parameters. In contrast, Students T-test was performed for CAT, APX, and SOD estimation experiments. The analysis included two independent variables: treatment type (a row factor) and experimental condition (a column factor), and their interaction. A percentage contribution of each factor as a source of variation was calculated, and a significance level of p ≤ 0.05 was established. All statistical analyses were performed using GraphPad Prism, and the effect size was reported as mean ± standard deviation (SD) of at least three biological replicates.

## 5 Results and discussion

### 5.1 Physio-chemical characteristic features of IAA- and IBA stabilised gold nanoparticles

**Figure 1:**
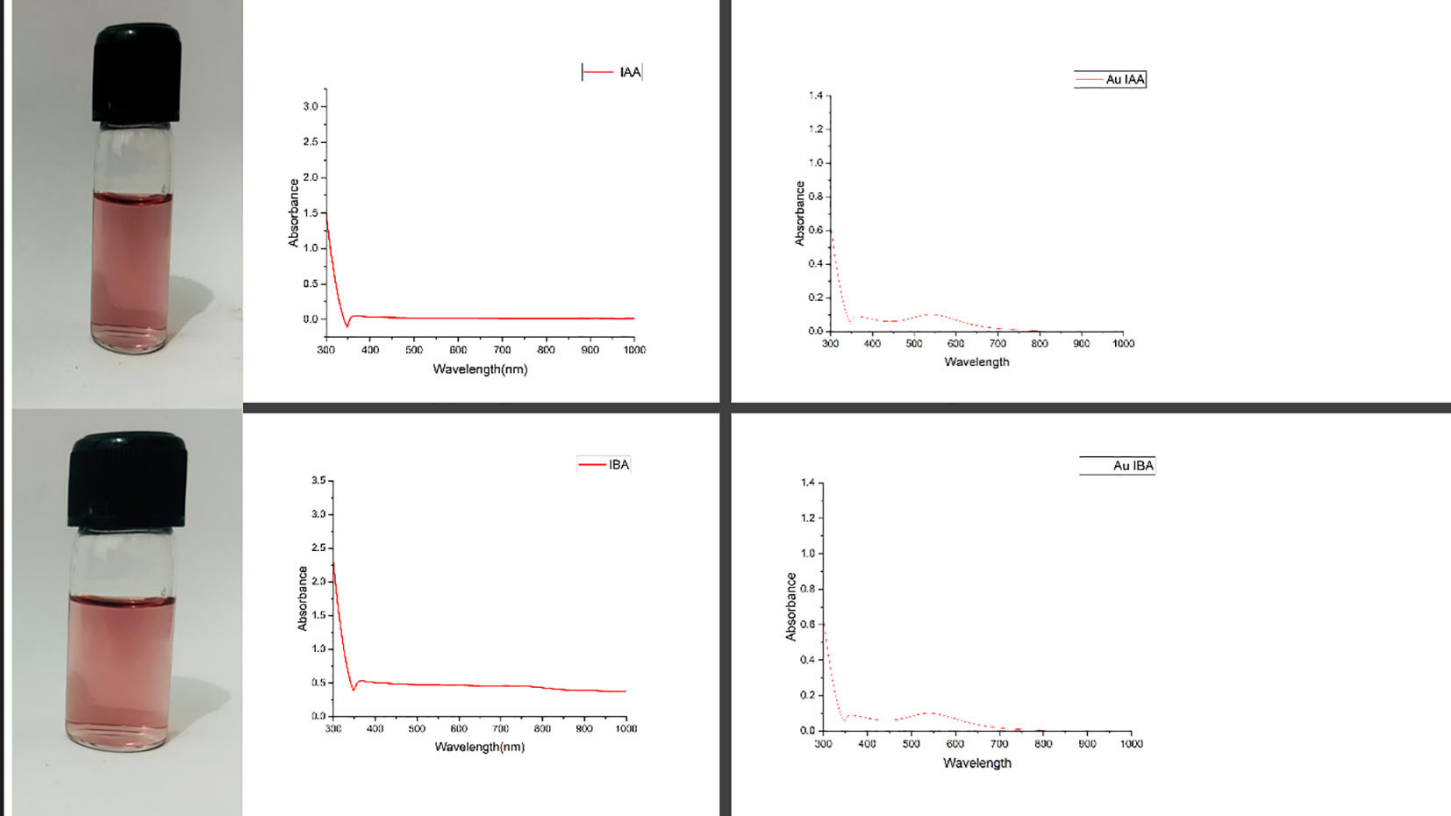
UV-visible excitation spectral data for IAA, IBA, AuIAA and AuIBA.

The wine-red gold colloids synthesised using IAA and IBA through a heat-mediated wet chemical reduction method exhibited characteristic UV-Vis absorption spectra, as illustrated in **Figure 1**. An absorption band with a maximum between 520-540 nm indicates spherical or near-spherical nanoparticles, a hallmark of successful nanoparticle formation. This spectral band, known as the surface plasmon resonance (SPR), confirms the reduction of HAuCl_4_ to gold nanoparticles (AuNPs) by IAA and IBA, as documented in previous studies (Maciej Serda et al., 2013). Both IAA and IBA stabilised AuNPs colloids displayed absorbance maxima (λ_max_) beyond 300 nm, implying a range of particle sizes, from smaller to larger nanoparticles, likely influenced by the concentration of the respective rooting hormones. The SPR bands observed in the UV-Vis spectra substantiate the effective use of IAA and IBA as reducing agents in synthesising AuNPs. The broad absorption maxima beyond 300 nm could be attributed to a polydisperse nature of the nanoparticles, suggesting that different particle sizes may form depending on the concentration of IAA and IBA (Ghosh et al., 2020). Higher concentrations of these indole compounds could potentially promote the formation of larger particles, while lower concentrations may favour smaller nanoparticles. This synthesis method leverages the unique properties of IAA and IBA as both reducing and stabilising agents, eliminating the need for additional capping agents (Dimkpa et al., 2012). The colloidal stability and particle size distribution are critical, as these factors influence the optical properties and potential applications of the AuNPs, such as in catalysis, drug delivery, or bioimaging. Further studies might explore the effects of varying the concentrations and reaction conditions to fine-tune the nanoparticle sizes and enhance the desired optical properties for specific applications (Dheyab et al., 2022).

**Figure 2:**
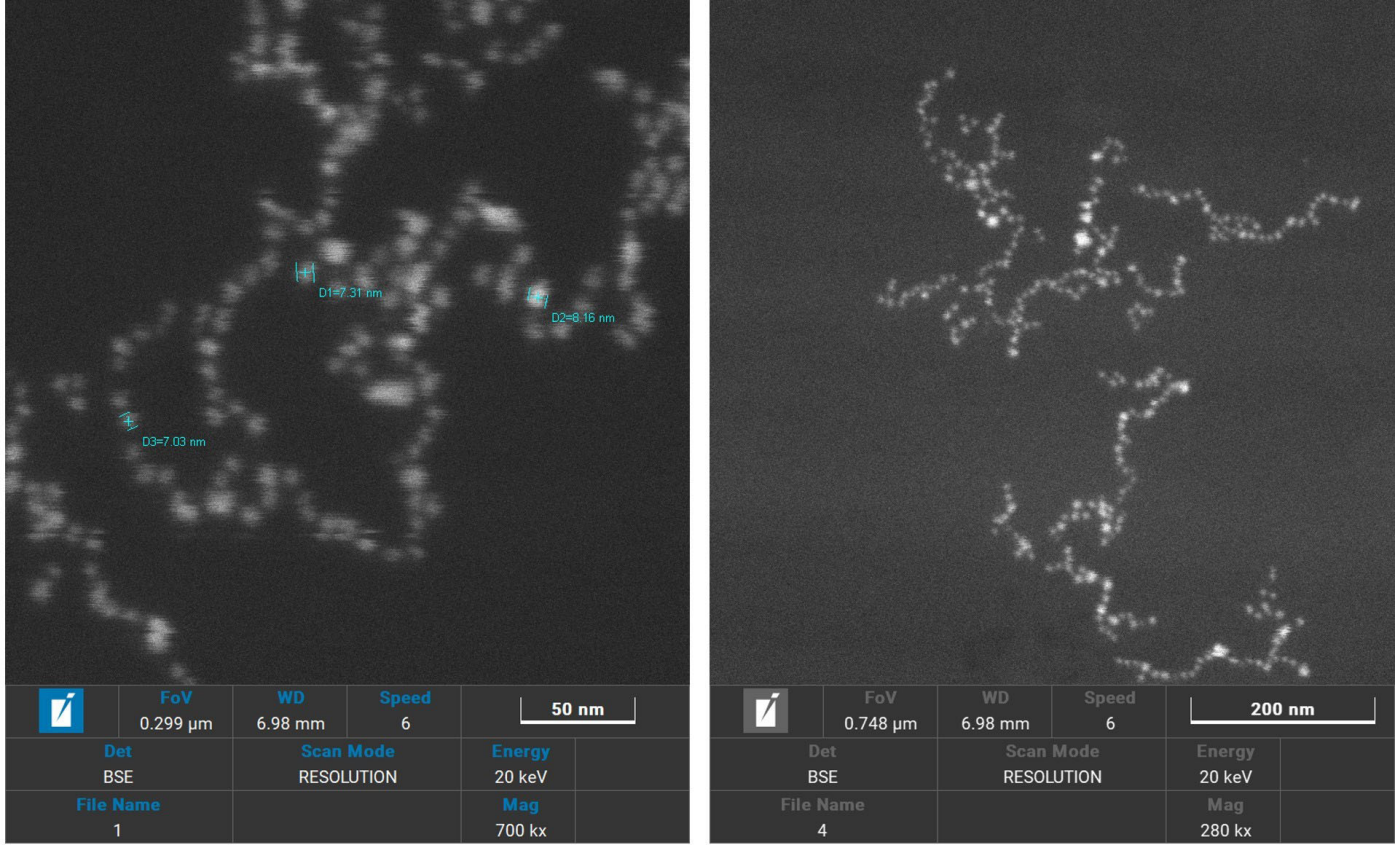
Scanning electron microscopy images of AuIAA and AuIBA at 200nm and 50 nm scales.

SEM was employed to analyse the morphology of the IAA and IBA stabilised AuNPs. It was observed that excessively concentrated samples reduced image quality, likely due to the increased contrast, impairing visualisation. Therefore, a diluted colloidal solution proved ideal for capturing high-quality micrographs with well-defined individual particles and enhanced resolution. Metal nanoparticles, such as those of silver, gold, copper, and aluminium, are readily observed via SEM due to their significantly higher electron densities compared to amorphous materials (Brännström et al., 2018). SEM images at 200 nm and 50 nm scales reveal uniformly shaped spherical nanoparticles stabilised by IAA and IBA (**Figure 2**). The AuNPs stabilised with IBA ranged in size from 6 to 70 nm, while those stabilised with IAA exhibited a broader size distribution, from 20 to 150 nm. The characteristic red-wine colour of the colloidal solution was indicative of the presence of gold nanoparticles in the 20 to 150 nm range, as confirmed by SEM observations (Pan et al., 2019) And the DLS graph. The SEM analysis provided insight into the morphological characteristics of AuNPs stabilised with IAA and IBA. Observations confirmed that sample concentration directly impacts SEM image quality, emphasising the importance of dilution for capturing distinct, high-resolution images (Goldstein et al., 2014). Additionally, the SEM images revealed that IBA-stabilised nanoparticles demonstrated a narrower size distribution compared to IAA-stabilised ones, possibly due to differences in molecular interactions between each stabilising agent and the gold nanoparticle surface. The narrower size range of IBA-stabilised AuNPs suggests more uniform particle stabilisation, which could be attributed to the steric and electronic effects of IBAs molecular structure. The broader distribution in IAA-stabilised nanoparticles may indicate more variable nucleation and growth conditions, which could be helpful in applications requiring a diverse size range (Ibrahim et al., 2019). Furthermore, the characteristic red-wine colour of the colloidal solution aligns with typical colourimetric properties associated with gold nanoparticle dispersions, reinforcing SEM and DLS findings. These morphological insights are crucial for optimising nanoparticle synthesis and tailoring particle size distributions for specific applications in biomedical and optical fields (Domingues et al., 2023).

**Figure 3:**
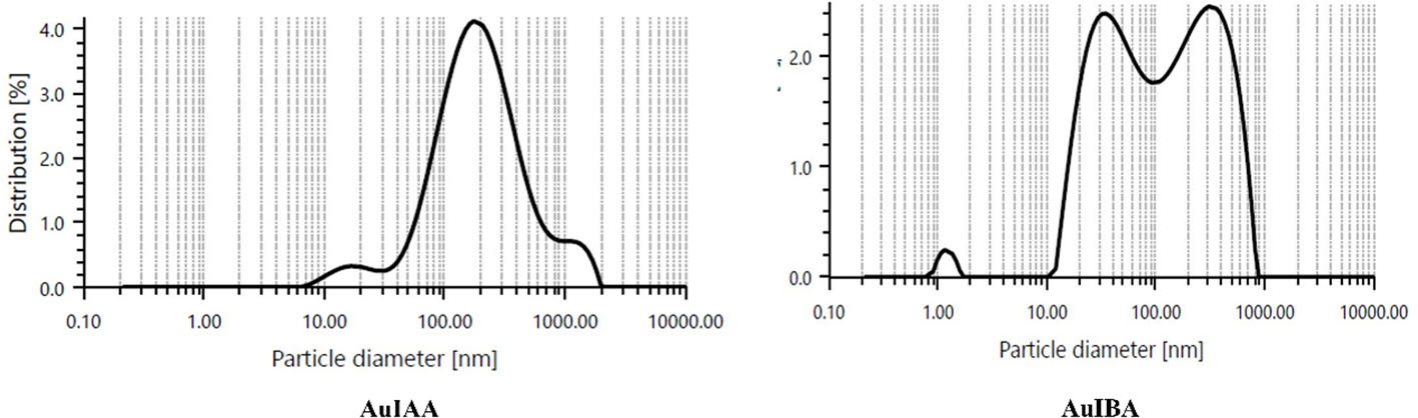
Dynamic light scattering graph of particle size distribution corresponding to AuIAA and AuIBA.

Dynamic light scattering (DLS) analysis indicated that the IAA stabilised AuNPs had a particle size distribution ranging from 10 to 1000 nm, with an average diameter of 188.03 nm (**Figure 3**). Meanwhile, IBA-stabilised AuNPs exhibited particle sizes between 10 and 100 nm, with an average diameter of 364 nm. Both colloidal solutions contained multiple populations of spherical particles of varying diameters, as reflected in the DLS histograms (Zheng et al., 2016). The differences in particle size distributions and average diameters between the IAA- and IBA-stabilised AuNPs suggest distinct stabilisation mechanisms and aggregation behaviours influenced by the respective stabilising agents. The broader size range observed in IAA-stabilised AuNPs could be due to less efficient stabilisation, allowing for more extensive particle growth or aggregation (Zhang et al., 2009). In contrast, IBA appears to stabilise smaller particle populations more consistently, yet results in a larger average diameter, which may indicate a preference for stabilisation of aggregated forms. The presence of multiple populations in both systems highlights the heterogeneity of particle formation in colloidal solutions and could affect the overall stability, reactivity, and potential application of these nanoparticles (Ganesh et al., 2018).

**Figure 4:**
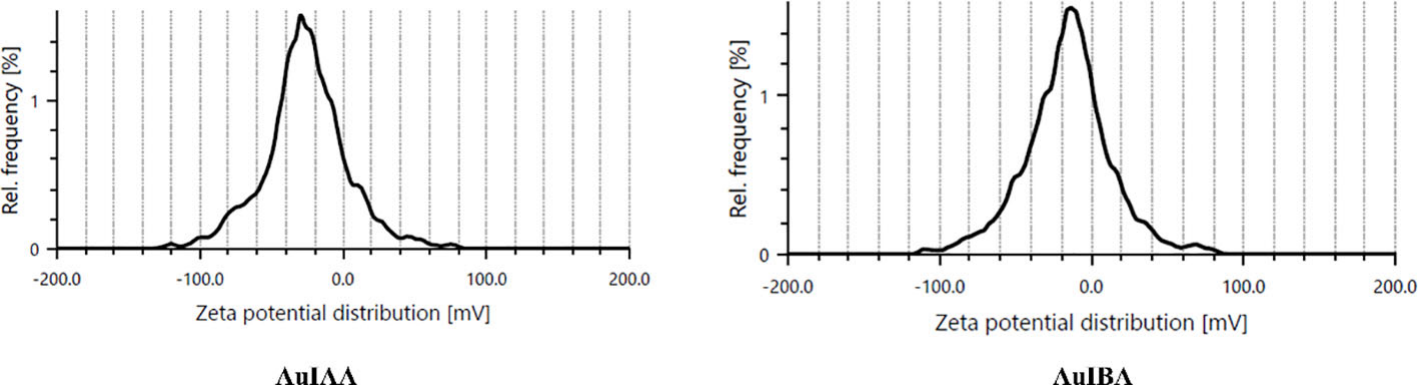
Zeta potential graph of particle charge distribution corresponding to AuIAA and AuIBA.

Further analysis of the particle stability was conducted using zeta potential measurements with Anton Paar litesizer 500 equipment. The mean zeta potential for IAA-stabilised AuNPs was recorded at approximately -28.2 mV, while IBA-stabilised AuNPs showed a slightly higher value of -28.9 mV (**Figure 4**). These negative surface charges produced electrostatic repulsion between the particles, promoting colloidal stability. A zeta potential greater than ±25 mV generally indicates highly stable nanoparticle dispersions. Indeed, the hormone-stabilised AuNPs demonstrated excellent stability, remaining stable for up to six months under room temperature conditions, as verified by UV-Vis spectra. The zeta potential results offer valuable insights into the stability of IAA- and IBA-stabilised AuNPs. With zeta potentials of +28.2 mV and -28.9 mV, respectively, both nanoparticle types exceed the ±25 mV threshold commonly associated with stable colloidal dispersions. The slightly higher stability of IBA-stabilised AuNPs suggests that IBAs structural properties may offer enhanced electrostatic repulsion (Thangavelu et al., 2018a). Comparable stability has been observed in other nanoparticles, such as chitosan-stabilised silver nanoparticles with a +40 mV zeta potential, stable cerium oxide nanoparticles around -30 mV, and silica nanoparticles with -50 mV, widely used in drug delivery. These examples highlight the importance of zeta potential in maintaining nanoparticle stability, underscoring the effectiveness of IAA and IBA as stabilising agents (Schlinkert et al., 2015).

**Figure 5:**
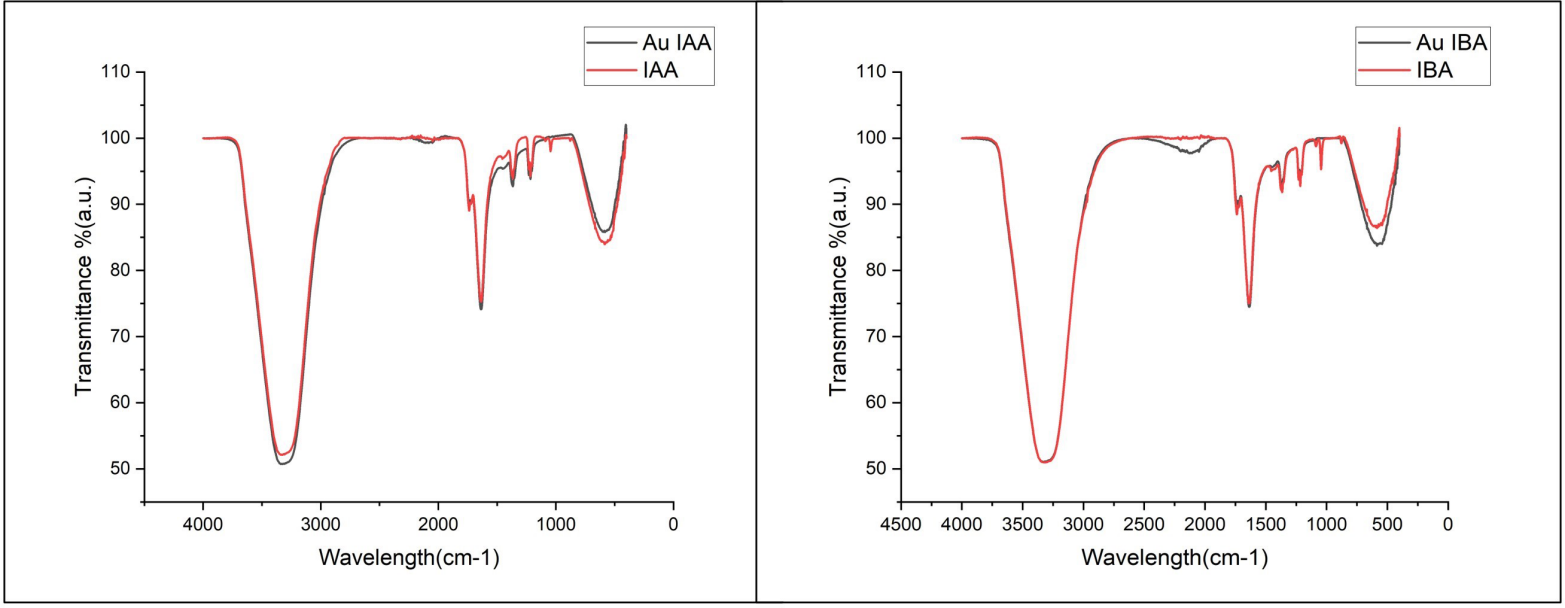
FTIR spectra of IAA and Au IAA. Key peaks at ~3300 cm^-^¹ (O-H/N-H stretching) and ~1700 cm^-^¹ (C=O stretching) show slight shifts and increased intensity in the Au IAA spectrum, indicating interaction between IAAs functional groups and Au nanoparticles and FTIR spectra of IBA and Au IBA. Peaks at ~3300 cm^-^¹ (O-H/N-H stretching) and ~1700 cm^-^¹ (C=O stretching) display minor shifts with increased intensity in the Au IBA spectrum, confirming the interaction of IBAs functional groups with Au nanoparticles.

To further investigate the interaction between IAA and IBA with the Gold nanoparticles, Fourier-transform infrared (FTIR) spectroscopy was conducted. This analysis provided insights into the functional groups that stabilise and reduce gold nanoparticles by the auxin hormones. The overlapping FTIR spectra of IAA and Au IAA are shown in **Figure 5**. Notable absorption peaks were observed around 3300 cm^-^¹, corresponding to stretching vibrations of the O-H or N-H, signifying the presence of amine or hydroxyl groups. Additionally, a prominent peak near 1700 cm^-^¹ was attributed to the carbonyl (C=O) group, suggesting the presence of carboxylic acids, esters, or ketones. Peaks between 1500 cm^-^¹ and 1000 cm^-^¹ were assigned to C-H bending and C-N stretching vibrations, indicating organic compounds within the samples. After introducing Au nanoparticles into IAA, the FTIR spectra displayed only minor shifts in peak positions, with slight variations in intensity. Specifically, the characteristic peaks around 3300 cm^-^¹ and 1700 cm^-^¹ showed minimal shifts, indicating that the interaction between IAA and the Au nanoparticles did not significantly alter the core molecular structure of IAA. These subtle shifts and slight increases in peak intensity suggest successful interaction between the functional groups of IAA and the Au nanoparticles, confirming the stabilisation of Au by IAA (Schlinkert et al., 2015).

Similarly, the Indole-3-butyric acid (IBA) FTIR spectrum revealed a broad absorption peak around 3300 cm^-^¹, corresponding to O-H or N-H stretching vibrations. The spectrum also showed distinct peaks near 1700 cm^-^¹, attributed to the carbonyl (C=O) group, and in the range of 1500-1000 cm^-^¹, associated with C-H bending and C-N stretching vibrations. Upon stabilisation of AuNPs with IBA, the FTIR transmittance peaks exhibited minor shifts, with a slight increase in intensity, particularly around 3300 cm^-^¹ and 1700 cm^-^¹, representing the O-H and C=O groups, respectively These shifts and enhanced intensities suggest successful interaction between the functional groups of IBA and the Au nanoparticles, indicating the involvement of IBAs hydroxyl and carbonyl groups in the capping and stabilization of the nanoparticles. Both IBA and IAA functioned as reducing agents and stabilizers, regulating nucleation and growth during the synthesis of AuNPs, as supported by similar findings in the literature (Joseph et al., 2022). Similar FTIR studies on nanoparticle systems have shown that organic compounds with hydroxyl and carbonyl groups are effective stabilisers. For instance, plant-extract-mediated synthesis of silver nanoparticles exhibited characteristic O-H and C=O peaks that confirmed effective capping and stability (Thatyana et al., 2023). In another study, chitosan-stabilised copper nanoparticles displayed broad O-H peaks and minor shifts in C-H bending regions, indicating stable capping interactions between chitosan and CuNPs (Muthukrishnan, 2015). This suggests that the interactions in IAA- and IBA-stabilised AuNPs follow similar trends, where organic functional groups (especially hydroxyl and carbonyl) contribute to effective nanoparticle capping and stabilisation.

### 5.2 Biochemical studies of seedlings of pearl millet

**Figure 6:**
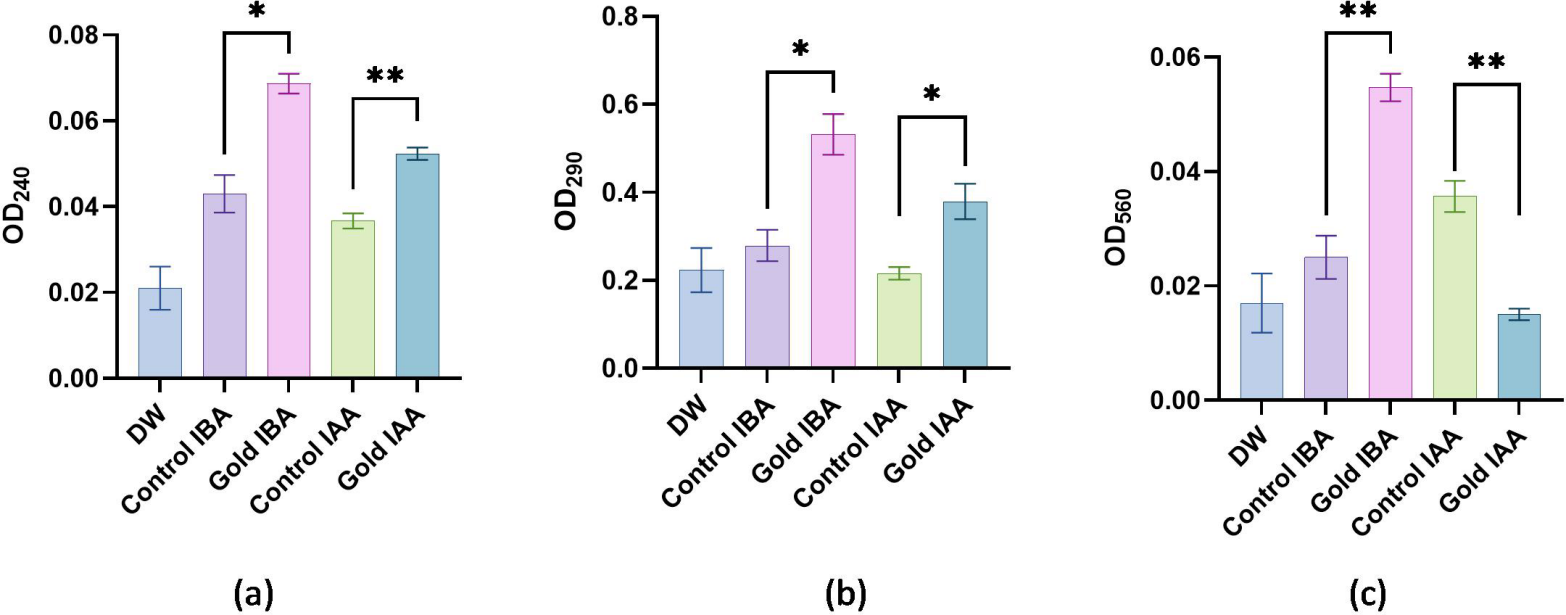
Biochemical activity: **(a)** Absorbance values representing catalase activity. **(b)**Absorbance values representing ascorbate peroxidase activity. **(c)** Absorbance values representing Superoxide Dismutase activity. "*": Shows significant difference between the growth. "**": Shows highly significant difference between the growth.

The biochemical evaluation was carried out, with the results noted (**Table 1**) and presented in **Figures 6a–c**. The catalase activity of the samples was assessed through absorbance measurements, with higher absorbance values indicating increased enzyme activity. Double-distilled water (DDW), used as the baseline control, exhibited the lowest absorbance value of 0.021, signifying minimal or negligible catalase activity without any active agents. The Control IBA sample showed a moderate increase in absorbance at 0.048, indicating a corresponding increase in catalase activity, but still relatively low compared to the nanoparticle-containing samples. The introduction of gold nanoparticles had a notable impact on catalase activity. The Gold IAA sample displayed an absorbance of 0.054, while Gold IBA showed a slightly higher absorbance at 0.057. These results suggest that gold nanoparticles enhance catalase activity, with Gold IBA demonstrating a marginally greater effect than Gold IAA. This enhancement in enzyme activity can be attributed to the catalytic properties of gold nanoparticles, which may promote more efficient catalase interactions (**Figure 6a**). Although the differences between the gold IAA and gold IBA samples are subtle, the results indicate a consistent trend: gold nanoparticles, especially those synthesised with IBA, contribute to an increase in catalase activity. These findings underscore the potential of gold nanoparticles to influence enzymatic reactions, offering insight into their potential applications in biocatalysis or biomedical fields where enzyme activity modulation is crucial.

**Table 1:** Catalase, ascorbate peroxidase, and superoxide dismutase activities in pearl millet samples treated with auxin-stabilized gold nanoparticles and controls.

CAT activity measured as absorbance (ABS (Mean ± SD)	
SAMPLE	ABS (Mean ± SD)
DW	0.021 ± 0.007118
Control IBA	0.043 ± 0.0061644
Gold IBA	0.068667 ± 0.0033
Control IAA	0.0366667 ± 0.0024944
Gold IAA	0.052333 ± 0.002055
APX activity measured as absorbance (ABS (Mean ± SD)	
SAMPLE	ABS (Mean ± SD)
DW	0.223333 ± 0.071336
Control IBA	0.2126667± 0.0115854
Gold IBA	0.362 ± 0.022686
Control IAA	0.296± 0.0857438
Gold IAA	0.429333 ± 0.123831
SOD activity measured as absorbance (ABS (Mean ± SD)	
SAMPLE	ABS (Mean ± SD)
DW	0.017± 0.007348
Control IBA	0.025± 0.0053541
Gold IBA	0.054667± 0.003399
Control IAA	0.0223333± 0.0033993
Gold IAA	0.045333± 0.008576

Values are expressed as mean absorbance (± SD).

The APX activity of the samples was analysed by measuring their absorbance, with higher absorbance values indicating increased enzyme activity. The DDW used as a baseline control exhibited the lowest absorbance value of 0.223, reflecting minimal APX activity without active agents. The Control IBA sample showed a notable increase in absorbance to 0.346, suggesting moderate enzyme activity, likely due to the presence of IBA, which enhances APX function compared to the baseline. The introduction of gold nanoparticles significantly boosted APX activity. The Gold IAA sample recorded an absorbance of 0.396, marking an apparent increase in enzyme activity over the Control IBA. This indicates that gold nanoparticles, in combination with IAA, enhance the catalytic efficiency of the APX enzyme. Gold IBA demonstrated an even greater effect, with the highest absorbance value of 0.462. This suggests that the combination of gold nanoparticles and IBA produces a more pronounced APX activity enhancement than the other samples (**Figure 6b**). These results reveal that gold nanoparticles, particularly in the gold IBA sample, substantially elevate APX activity, likely due to their unique catalytic properties. This finding underscores the potential role of gold nanoparticles in enhancing antioxidant enzyme function, which could have significant implications for biomedical applications where the modulation of oxidative stress and enzyme activity is critical.

The SOD activity of the samples was evaluated through absorbance measurements, where higher absorbance values indicate increased enzymatic activity. The DDW sample, serving as the baseline, showed the lowest absorbance at 0.017, reflecting minimal SOD activity in the absence of any active agents. The Control IBA sample demonstrated a slight increase in absorbance to 0.023, suggesting a moderate improvement in SOD activity compared to the baseline. The introduction of gold nanoparticles further enhanced SOD activity. The Gold IAA sample exhibited an absorbance of 0.029, indicating a significant increase in enzyme activity compared to the Control IBA sample. This suggests that the presence of gold nanoparticles, in conjunction with IAA, boosts the antioxidant capacity of SOD. The highest SOD activity was observed in the gold IBA sample, with an absorbance of 0.032, demonstrating that gold nanoparticles synthesised with IBA promote the most substantial enhancement of SOD activity among the tested samples (**Figure 6c**). These results suggest that gold nanoparticles, particularly in the gold IBA sample, effectively enhance SOD activity. The ability of gold nanoparticles to increase the efficiency of antioxidant enzymes like SOD highlights their potential for applications in oxidative stress regulation, which could be beneficial in fields such as biomedicine and therapeutics.

The results indicate that gold nanoparticles enhance the activity of key antioxidant enzymes CAT, APX and SOD compared to baseline and untreated controls. Gold nanoparticles synthesised with IBA showed the highest activity across all enzymes, suggesting that IBA provides superior stabilisation and catalytic enhancement. The increased enzyme activity in the presence of gold nanoparticles highlights their potential to modulate antioxidant responses, making them promising candidates for applications in biocatalysis and biomedical fields where oxidative stress management is essential (Pizzino et al., 2017).

### 5.3 Effects of gold nanoparticles synthesised using IAA and IBA on pearl millet seed growth

**Figure 7:**
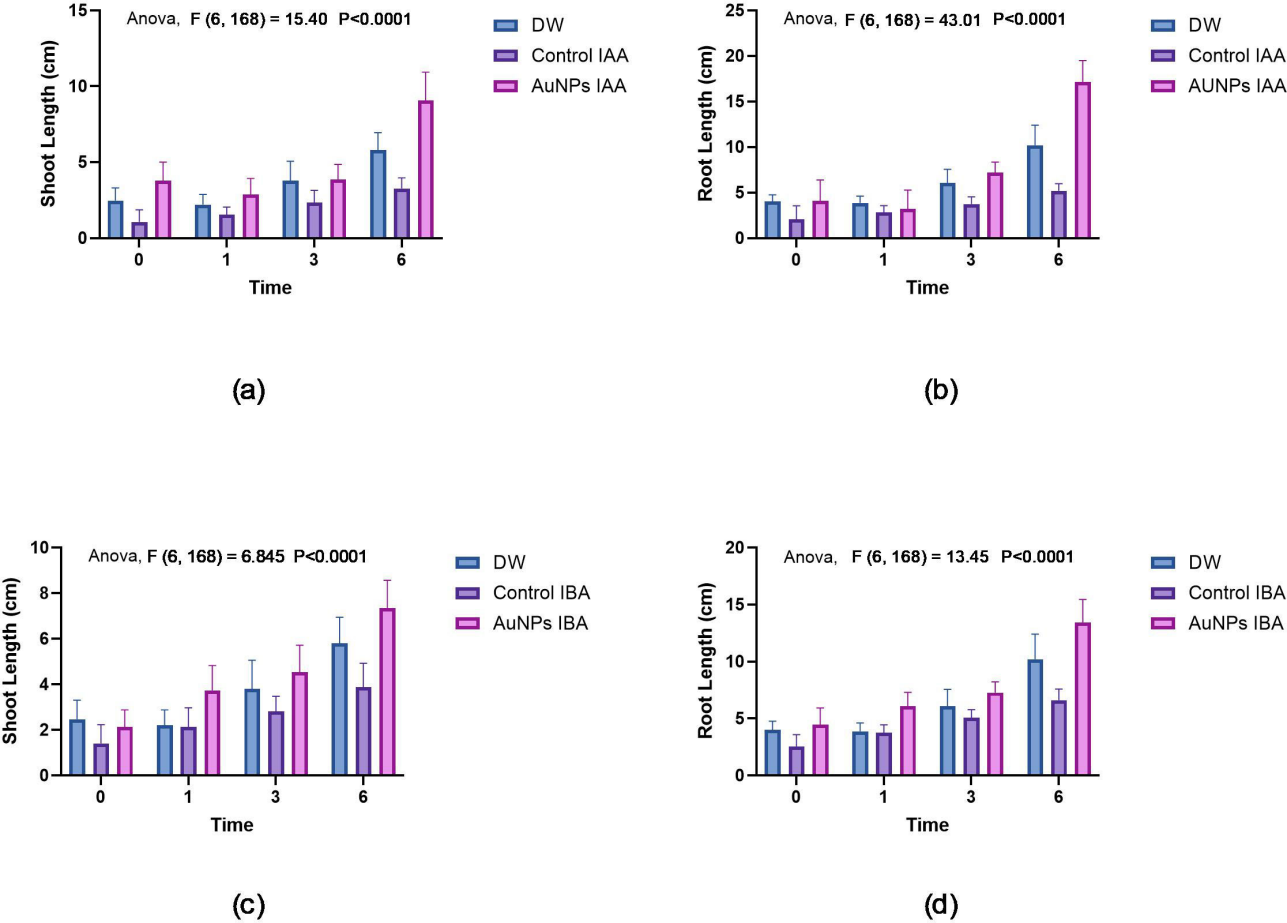
Growth of pearl millet seeds: **(a)** Shoot lengths of pearl millet seeds treated with gold nanoparticles synthesised by IAA. **(b)** Root lengths of pearl millet seeds treated with gold nanoparticles synthesised by IAA. **(c)** Shoot lengths of pearl millet seeds treated with gold nanoparticles synthesised by IBA. **(d)** Root lengths of pearl millet seeds treated with gold nanoparticles synthesised by IBA.

The growth performances of pearl millet seeds (untreated and treated) with IAA AuNPs and IBA AuNPs were assessed through their associated shoot and root lengths. There was little growth in the control of untreated seeds, with shoot lengths averaging 2 cm and root lengths averaging 3.5 cm. On the other hand, the seeds of IAA AuNP-treated seeds had increased shoot lengths of 5.25 cm and root length of 6.75 cm (**Figures 7a, b** **).** Although IBA AuNP-treated seeds had less shoot growth 4.75 cm than IAA AuNP-treated seeds, they had greater root length growth 7.75 cm (**Figures 7c, d** **).**

**Figure 8:**
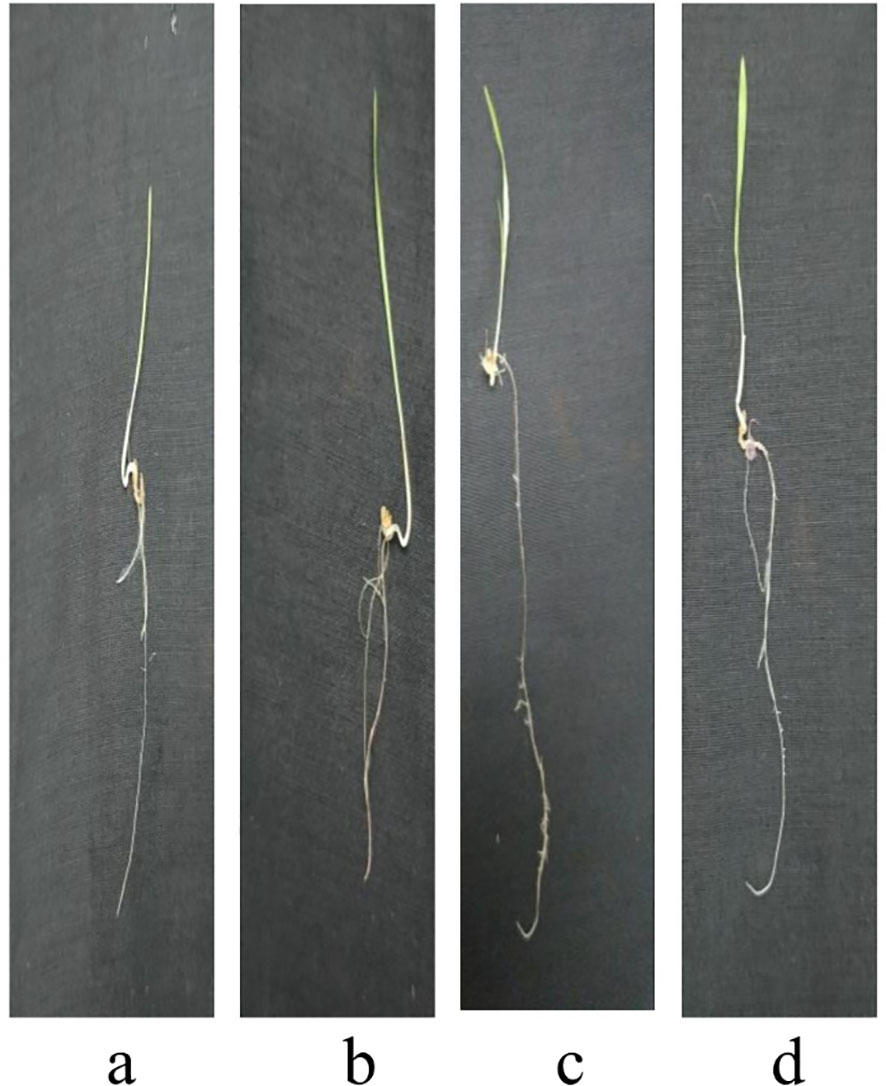
Growth of plants after seed priming with AuNPs IAA **(a)** 0 hr **(b)** 1 hr **(c)** 3 hr **(d)** 6 hr.

**Figure 9:**
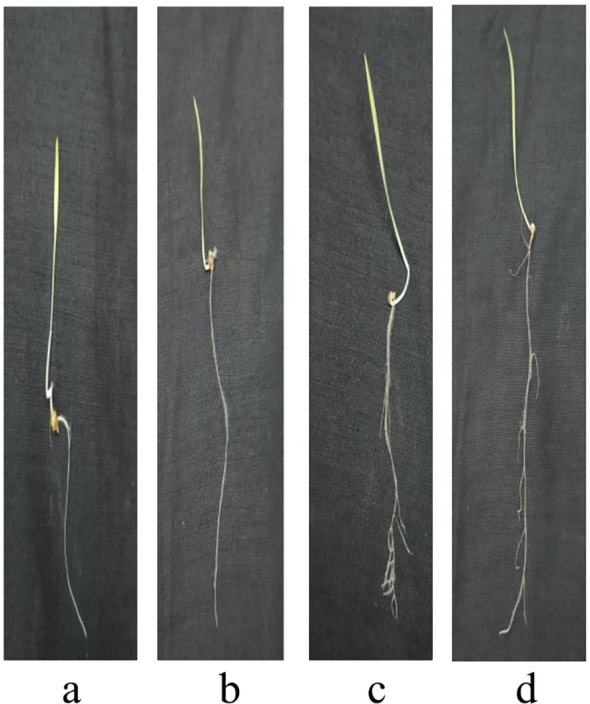
Growth of plants after seed priming with AuNPs IBA **(a)** 0 hr **(b)** 1 hr **(c)** 3 hr **(d)** 6 hr.

**Figure 10:**
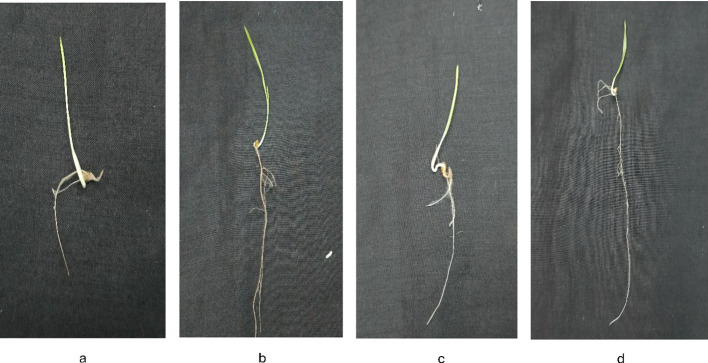
Growth of plants after seed priming with control IAA **(a)** 0 hr **(b)** 1 hr **(c)** 3 hr **(d)** 6 hr.

**Figure 11:**
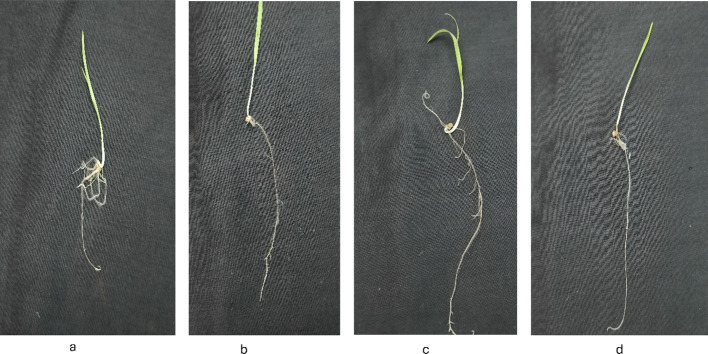
Growth of plants after seed priming with control IBA **(a)** 0 hr **(b)** 1 hr **(c)** 3 hr **(d)** 6 hr.

**Figure 12:**
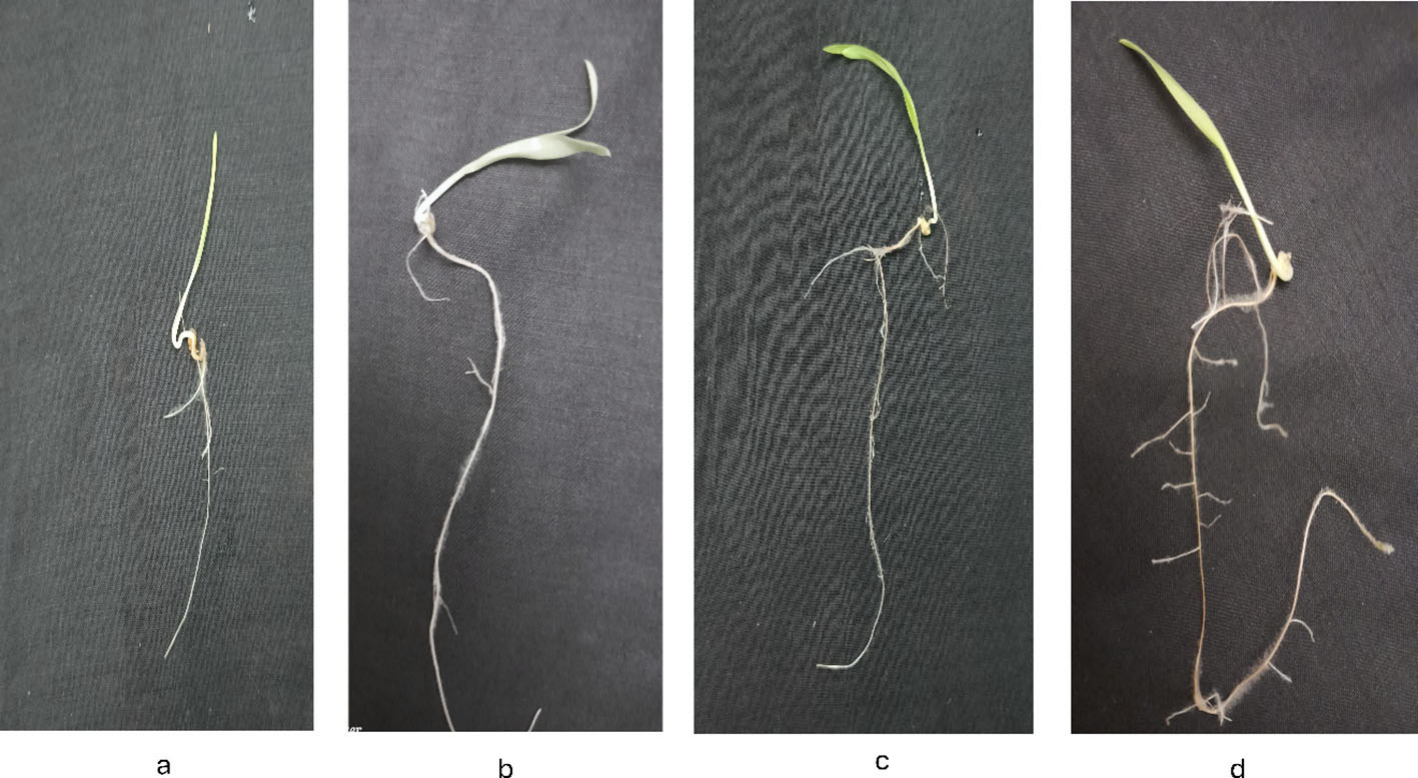
Growth of plants after seed priming with milli-q water **(a)** 0 hr **(b)** 1 hr **(c)** 3 hr **(d)** 6 hr.

**Figures 8**-**12** provide additional visual comparisons to support these results. **Figures 8**, **9** portray plant development after seed priming with AuNPs (synthesised using IAA and IBA, respectively) at 0, 1, 3, and 6 hours. **Figures 10**, **11** show the plants stage after seed priming with the same control IAA and IBA solutions (**Table 2**). **Figure 12** shows the stage of plants due to being treated with Milli-Q water. Overall, it is evident that both IAA- and IBA-stabilised gold nanoparticles influenced spontaneous shoot and root development in pearl millet. While the IAA-treated AuNPs appeared to be more efficient relative to shoot elongation, the IBA-stabilised AuNPs were more efficient in promoting root development. These were strong indicators or testimony to the potential of using auxin-stabilised gold nanoparticles to act as environmentally friendly and efficient plant growth promoters in agriculture.

**Table 2:** Root and shoot lengths (Mean ± SD) of pearl millet seedlings over time (0–6 hours) under different treatments, including Milli-Q water control auxins (IAA/IBA), and AuNPs-stabilized IAA and IBA.

S.No	Treatment	Time/Duration (h)	Root length (cm) Mean ± SD	Shoot length (cm) Mean ± SD
1	DW	0.0	04.0 ± 0.75	2.47 ± 0.83
		1.0	3.86 ± 0.74	2.2 ± 0.67
		3.0	6.06 ± 1.48	3.8 ± 1.26
		6.0	10.2 ± 2.21	5.8 ± 1.14
2	Control IAA	0.0	2.06 ± 1.48	1.06 ± 0.79
		1.0	02.8 ± 0.77	1.53 ± 0.51
		3.0	3.73 ± 0.79	2.33 ± 0.81
		6.0	5.2 ± 0.77	3.26 ± 0.70
3	AuNPs IAA	0.0	4.06 ± 2.31	3.8 ± 1.16
		1.0	3.2 ± 2.00	2.8 ± 1.06
		3.0	7.2 ± 1.14	3.86 ± 0.99
		6.0	17.13 ± 2.35	9.06 ± 1.86
4	Control IBA	0.0	2.53 ± 1.06	01.4 ± 0.82
		1.0	3.73 ± 0.70	2.13 ± 0.83
		3.0	5.06 ± 0.70	02.8 ± 0.67
		6.0	06.6 ± 0.98	3.86 ± 1.06
5	AuNPs IBA	0.0	4.46 ± 1.45	2.13 ± 0.74
		1.0	6.06 ± 1.22	3.73 ± 1.09
		3.0	7.26 ± 0.96	4.53 ± 1.18
		6.0	13.4 ± 2.06	7.33 ± 1.23

To statistically test the observed outcomes, a two-way ANOVA was conducted to evaluate the effects of treatment (row factor), experimental condition (column factor) and the interaction between treatment and condition on root and shoot lengths. The analysis results demonstrated that all sources of variation were significantly different (p < 0.0001) between treatment groups and are summarised in **Table 3**. The row factor (treatment) contributed the most to total variation in root and shoot growth for the IAA treatments, followed by the column factor and the interaction effects. For IBA plants, the treatment (row factor) provided the most total variation for roots and shoots. The interaction effect was comparatively smaller, but still significant, suggesting that the type of nanoparticle treatment and the various conditions under which they were applied both play essential roles in influencing the growth of the pearl millet plants.

**Table 3:** Two-way ANOVA results showing the percentage of total variation, significance levels (*p* < 0.0001), and interaction effects for root and shoot growth in pearl millet plants treated with IAA- and IBA-stabilized gold nanoparticles.

Two-way Anova of Gold IAA Root				
Source of Variation	% of total variation	P value	P value summary	Significant
Interaction	18.68	<0.0001	****	Yes
Row Factor	51.08	<0.0001	****	Yes
Column Factor	18.07	<0.0001	****	Yes
Two-way Anova of Gold IBA Root				
Source of Variation	% of total variation	P value	P value summary	Significant
Interaction	7.390	<0.0001	****	Yes
Row Factor	59.15	<0.0001	****	Yes
Column Factor	18.07	<0.0001	****	Yes
Two-way Anova of Gold IAA Shoot				
Source of Variation	% of total variation	P value	P value summary	Significant
Interaction	10.70	<0.0001	****	Yes
Row Factor	44.13	<0.0001	****	Yes
Column Factor	25.71	<0.0001	****	Yes
Two-way Anova of Gold IBA Shoot				
Source of Variation	% of total variation	P value	P value summary	Significant
Interaction	6.126	<0.0001	****	Yes
Row Factor	52.53	<0.0001	****	Yes
Column Factor	16.28	<0.0001	****	Yes

This study presents a novel and environmentally sustainable approach to enhancing the growth of pearl millet by synthesising gold nanoparticles (AuNPs) mediated by auxin, a key plant growth hormone. The integration of auxin, known for its role in promoting cell elongation, root formation, and overall development, with AuNPs, provides a significant boost in shoot and root biomass, length, and plant vigour (Roychoudhry and Kepinski, 2022; Singh et al., 2024)This growth enhancement likely results from the synergistic effects of auxin and AuNPs, which together stimulate cell division, improve nutrient assimilation, and enhance water absorption (Khadr et al., 2020). The AuNPs likely act as carriers for auxin, increasing its bioavailability and transport efficiency within plant tissues, which in turn results in a sustained hormonal effect on root and shoot development (Sosnowski et al, 2023). Additionally, the unique properties of AuNPs—such as their high stability, biocompatibility, and ability to interact effectively with plant cell membranes—make them particularly suitable for applications in plant growth (Qiao and Qi, 2021). These characteristics differentiate AuNPs from other nanoparticles, such as silver or zinc oxide, which have shown varying and sometimes inhibitory effects on plant growth in previous studies (Ehsan et al., 2022).

To build upon these promising findings, future research could delve into the longer-term impacts of auxin-mediated AuNPs on pearl millet yield, nutrient density, and resilience to abiotic stresses such as drought or salinity. Assessing these parameters would provide a more comprehensive understanding of the field-level applicability of this approach (Satyavathi et al., 2021; Soni et al., 2024). Additionally, investigating the interactions of AuNPs with other phytohormones like gibberellins or cytokinins could reveal even broader growth-promoting synergies, potentially enabling enhanced optimisation of plant development and resilience across diverse agricultural contexts (Shelar et al., 2024)

This research demonstrates a new paradigm in agricultural biotechnology using nanotechnology tools (e.g., gold nanoparticles and nanomaterials) with the natural plant growth-promoting properties of the phytohormones IAA and IBA. Ultimately, while previous studies reported the use of auxins as stabilisers in gold nanoparticles, to date, no one has reported using IAA and IBA as both reducing agents and stabilisers in the formation of gold nanoparticles, making this gold-aauxin nanoparticle fabrication unique, new, and highly relevant (2018b). Significantly, we characterised the auxin-mediated gold nanoparticles, particularly those that can dramatically facilitate better shoot and root growth in pearl millet, an essential, drought-tolerant crop. Additionally, our green chemistry method does not contain toxic chemicals, is environmentally applicable, green and sustainable, and uses the properties of the IAA and IBA as plasma materials that have physical and biological compatibility with the plant growth, reducing the chance of any phytotoxicity with the phytohormones as plant growth regulators (Yadav et al., 2022). On a larger scale by increasing plant resilience to stressors, and increased growth and yield in the face of limited resource availability and abiotic and biotic stressors, this approach has potential to contribute to more secure and sustainable food systems and sustainable agricultural practices that enhance food security through resilience to climate change, and as a viable alternative to chemical agrochemicals.

## 6 Conclusion

Using hormone-stabilised AuNPs offers a promising approach for promoting root development in pearl millet, a crucial crop in arid and semi-arid regions. The successful synthesis of AuNPs using auxin hormones, such as IAA and IBA, alongside their comprehensive characterisation using advanced analytical techniques, highlights the potential of these nanoparticles. *In vitro* studies utilising hydration priming demonstrated a remarkable threefold increase in root growth, underscoring the effectiveness of AuNPs in fostering robust root systems. Moreover, the absence of phytotoxicity in biochemical analyses further validates the safety of this method for agricultural use. These findings position AuNPs as a cutting-edge solution for enhancing crop resilience and productivity, offering a valuable tool for improving food security in regions where pearl millet is a staple crop. Further research is recommended to explore the broader applicability of this innovative approach to other crops and diverse environmental conditions.

## Data Availability

The raw data supporting the conclusions of this article will be made available by the authors, without undue reservation.
